# An Assessment of Serum Selenium Concentration in Women with Endometrial Cancer

**DOI:** 10.3390/nu14050958

**Published:** 2022-02-24

**Authors:** Magdalena Janowska, Natalia Potocka, Sylwia Paszek, Marzena Skrzypa, Andrzej Wróbel, Marta Kluz, Piotr Baszuk, Wojciech Marciniak, Jacek Gronwald, Jan Lubiński, Izabela Zawlik, Tomasz Kluz

**Affiliations:** 1 Department of Gynecology, Gynecology Oncology and Obstetrics, Fryderyk Chopin University Hospital No. 1, 35-055 Rzeszow, Poland; gibala.magda@gmail.com; 2 Laboratory of Molecular Biology, Centre for Innovative Research in Medical and Natural Sciences, Medical College of Rzeszow University, 35-959 Rzeszow, Poland; npotocka@ur.edu.pl (N.P.); sylwia.paszek@wp.pl (S.P.); mskrzypa@ur.edu.pl (M.S.); izazawlik@yahoo.com (I.Z.); 3 Second Department of Gynecology, Medical University of Lublin, 20-090 Lublin, Poland; wrobelandrzej@yahoo.com; 4 Department of Pathology, Fryderyk Chopin University Hospital No. 1, 35-055 Rzeszow, Poland; marta.kluz@interia.pl; 5 Department of Genetics and Pathology, Pomeranian Medical University, 70-204 Szczecin, Poland; baszukpiotr@gmail.com (P.B.); wojciech.marciniak90@gmail.com (W.M.); jgron@pum.edu.pl (J.G.); lubinski@pum.edu.pl (J.L.); 6 Institute of Medical Sciences, Medical College of Rzeszow University, 35-959 Rzeszow, Poland

**Keywords:** selenium, trace element, endometrial cancer, uterine cancer, serum Se level, microelements and endometrial cancer, cancer prevention

## Abstract

Background: Numerous studies have shown a relationship between low serum selenium levels and an increased risk of developing cancer. Methods: A total of 306 women participated in the study: 153 patients diagnosed with endometrial cancer and 153 healthy women who were matched, in terms of birth year (+/−3 years), to the patients from the study group. The quantitative measurement of selenium content in the collected blood samples was performed using a mass spectrometer with excitation in inductively coupled plasma. In order to determine the relationship between the risk factors and the incidence of endometrial cancer, analyses based on single- and multi-factor conditional logistic regression models were performed. Results: The mean concentration of selenium was lower in patients with endometrial cancer than in healthy controls (60.63 µg/L (0.77 µmol/L) vs. 78.74 µg/L (0.99 µmol/L), respectively). When compared in quartiles, a significant association of lower selenium concentration with the incidence of endometrial cancer was recorded. The highest OR was observed in the first and second quartiles (OR-22.0, *p*-value < 0.001; medium selenium level 46.95 µg/L (0.59 µmol/L), and OR-5.94; *p*-value < 0.001; medium selenium level 63.60 µg/L (0.80 µmol/L), respectively). Conclusion: A strong correlation between the level of selenium in the blood serum and the risk of endometrial cancer indicates that patients with low levels should be a candidate group requiring appropriate preventive examinations. Further research on a larger group of patients is required.

## 1. Introduction

Selenium is a trace element necessary for the proper functioning of the human body. Selenium is required for the formation of selenocysteine, an amino acid that is genetically encoded and is required for the formation of a range of transcribed proteins known as selenoproteins. This element shows a protective function against free radicals due to its presence in active centers of antioxidant enzymes, such as glutathione peroxidase [1,2,3]. By affecting the immune system, selenium increases the activity of immune cells [4]. In the diet, selenium is found mainly in cereals, vegetables, seafood, meat, dairy products, and nuts [5]. This element is supplied to the human body through food or dietary supplements in two forms: organic (selenomethionine and selenocysteine) and inorganic (selenites and selenates). Both of these forms are transformed into selenides, which are directly involved in the formation of selenoproteins [6]. According to the recommendations of the World Health Organization (WHO), the minimal daily dose of selenium is 55 µg for adults [7]. The American Food and Nutrition Council recommends a daily dose of selenium: 40–70 µg for men and 45–55 µg for women [8].

Endometrial cancer is the most common neoplasm of the female reproductive system [9]. In 2020, 417,367 new cases of endometrial cancer were registered worldwide, and 97,370 deaths were recorded. For comparison, in 2018 there were 37,367 fewer new diagnoses of endometrial cancer compared with the 2020 data [10].

The main risk factors for the development of endometrial cancer include: increasing age, obesity, insulin resistance, weight gain in adulthood, increased waist-to-hip ratio, and the coexistence of chronic diseases such as hypertension and diabetes. Factors favoring the development of endometrial cancer include: polycystic ovary syndrome, early first menstruation, late menopause, non-fertility, unopposed estrogen hormone replacement therapy, no-ovulation cycles, and no breastfeeding. Genetic factors include Lynch syndrome, Cowden syndrome, and a current family history of endometrial cancer and colorectal cancer. Iatrogenic factors include tamoxifen therapy [11,12,13].

Numerous publications have shown a correlation between the level of selenium in human serum and the incidence effect on cancer. Such correlations have been studied, among others, for colon, lung, bladder, breast, ovary, and prostate cancers. Therefore, this element is becoming more and more popular among scientists [14,15,16,17,18,19,20,21]. A relatively small number of studies show a correlation between serum selenium levels and the risk of developing endometrial cancer. Our study aimed to investigate the relationship between serum selenium concentrations and the risk of developing endometrial cancer.

The relationship between the level of various micronutrients and the development of endometrial cancer is of increasing interest among scientists. Among other elements examined, the following should be mentioned: copper, zinc, iron, and cadmium. The results obtained, so far, do not show a clear correlation between the levels of these elements in the female body and the development of endometrial cancer [22,23,24,25].

## 2. Materials and Methods

The study involved 153 patients diagnosed with endometrial cancer. All diagnoses were confirmed by previous histopathological examinations. These patients reported to the Department of Gynecology and Clinical Obstetrics, Provincial Hospital No. 1 in Rzeszow, in 2016–2019 in order to start oncological treatment. Each patient had blood samples taken before the planned surgery. A healthy woman was matched to each patient from the study group, in terms of the year of birth (+/−3 years), in order to create a control group. All of the women agreed to have additional blood samples taken for the research. The research was carried out with the consent of the Bioethics Committee (Resolution No. 90/B/2016 of the Bioethics Committee of the Regional Medical Chamber of 24 November 2016). Additionally, both in the study group and in the control group, a questionnaire that took into account the risk factors for the development of endometrial cancer was conducted. Clinical and lifestyle characteristics of patients affected with endometrial cancer and healthy controls are shown in Table 1. These data are used to illustrate that the participants from the control and study groups were matched to each other in terms of as many factors as possible. No statistical significance was obtained for the following factors: age, menopausal status, taking contraception, endometriosis, hypertension, smoking, age of first menstruation, and hypothyroidism. The statistically significant factors include: hormone replacement therapy, diabetes, number of births, breastfeeding, and BMI. The influence of risk factors on the selenium level results is included in Table 2 through the use of multivariate conditional logistic regression.

### 2.1. Sample Collection and Storage

Sera were collected with the Sarstedt Monovette system (Sartedt, Germany) using Serum Z/7.5 mL tubes by a standard antecubital venipuncture. The collected serum was incubated at room temperature for at least 30 min, but for no longer than 2 h to avoid clotting, and then was centrifuged at 1300× *g* for 12 min. After that, the serum was transferred into cryovials and placed into the freezer at −80 °C. Patients’ sera were stored at −80 °C until the analysis. On the day of the analysis, sera were thawed, vortexed, and centrifuged at 5000× *g* for 5 min before selenium determination.

### 2.2. Measurement Methodology

Determination of selenium (^80^Se) was performed using an ICP mass spectrometer ELAN DRC-e (PerkinElmer). Before each analytical run, the instrument was tuned to achieve the manufacturers’ criteria. Oxygen was used as a reaction gas. Technical details are available on request. The spectrometer was calibrated using an external calibration technique. Calibration standards were prepared fresh daily, from 10 µg/mL Multi-Element Calibration Standard 3 (PerkinElmer, Waltham, MA, USA), by diluting with a blank reagent to the final concentration of 1; 2; 5; 10; 50 µg/L. Correlation coefficients for calibration curves were always greater than 0.999. To minimize matrix effects on the Se signal, the two approaches used were the matrix-matched calibration technique and the internal standard technique. Rhodium ^105^Rh was selected as the most appropriate internal standard. An analysis protocol assumed a 30-fold dilution of serum in a blank reagent. The blank reagent consisted of high-purity water (>18 MΩ), TMAH (AlfaAesar, Haverhill, MA, USA), Triton X-100 (PerkinElmer), n-butanol (Merck, Darmstadt, Germany), and EDTA (Sigma Aldrich, St. Louis, MI, USA).

### 2.3. Quality Control

Accuracy and precision of measurements were tested using certified reference material (CRM), Clincheck Plasmonorm Serum Trace Elements Level 1 (Recipe, Germany). Recovery rates for ^80^Se were between 96% and 110%; calculated precision (Cv%) was 6.04%. Method LOQ was calculated to be 0.113 µg/L. Additionally, both in the study group and in the control group, a questionnaire was carried out that took into account the risk factors for the development of endometrial cancer.

### 2.4. Statistics

The participants of the study were assigned to one of four equal groups (quarters), determined in relation to the increasing distribution of selenium concentrations. The concentration ranges of individual quarters are presented in Table 2. In order to determine the relationship of the risk factors with the incidence of endometrial cancer, some analyses were performed based on univariable and multivariable conditional logistic regression models (Table 2). The following factors were taken into account for the analyses: serum selenium level expressed in quarters, age, menopausal status, use of contraception, use of hormone replacement therapy, coexisting diseases (endometriosis, hypertension, diabetes, hypothyroidism), fertility, breastfeeding, age of first menstruation, body mass index (BMI), and smoking.

## 3. Results

The selenium levels for each quarter and risk factors of endometrial cancer are shown in Table 2. The average level of selenium for all participants of the study was 69.68 µg/L (8.85–170.76 µg/L; 0.88 µmol/L). Selenium obtained in the study group was at the level of 60.63 µg/L (8.85–164.42 µg/L; 0.77µmol/L), and in the control group was at the level of 78.74 µg/L (42.85–170.76 µg/L; 0.99 µmol/L). The level of selenium for the first quarter was 46.95 µg/L (8.85 µg/L −57.28 µg/L; 0.59 µmol/L). In total, 43% of the patients belonging to this quarter were female patients from the study group, and 7.2% were female patients from the control group. The OR was 22 (95% Cl 7.94–61.2) and statistically significant (*p*-value < 0.001). For the second quarter, the average selenium level was 63.60 µg/L (57.35–69.76 µg/L; 0.80 µmol/L). Among the 76 patients assigned to this quarter, 18% were patients from the control group and 31% were from the study group. The OR was at 5.94 (95% Cl 2.59–13.6). The *p*-value was <0.001. The level of selenium obtained for the third quarter was 75.05 µg/L (69.90–80.45 µg/L; 0.95 µmol/L). Of the patients belonging to this quarter, 51 were patients from the control group and 25 were from the study group. However, no statistical significance was achieved. In the fourth quarter, the average selenium level was 93.12 µg/L (80.64–170.76 µg/L; 1.18 µmol/L). Among the 77 women in this quarter, 63 (25%) belonged to the control group and 14 (9.2%) to the study group.

Using one- and multi-factor conditional logistic regressions, we show the impact of the risk factors for endometrial cancer that we take into account on the results presented by us. In the multivariate analysis, the OR for the selenium level in the first quadrant was 17.8 (95% Cl 5.20–59.0) and this result remained statistically significant (*p*-value < 0.001). The OR for the selenium level in the second quadrant changed from 5.94 to 6.40 (*p*-value < 0.001). In the third quadrant, the OR for the level of selenium in the multivariate analysis was obtained at the level of 1.42 (*p*-value < 0.5). In multivariate analyses, statistical significance remained only for two risk factors for the development of endometrial cancer: breastfeeding and BMI.

## 4. Discussion

There are few publications describing the relationship between the level of selenium in the blood serum and the risk of developing endometrial cancer. The analyses carried out, so far, do not provide a clear answer as the results vary. In 1984, H. Sundström et al. determined the level of selenium in the blood serum in 37 patients with cervical cancer and in 64 patients with endometrial cancer. The results were compared with the control group (137 women), obtaining a statistically significant (*p*-value < 0.001) lower selenium level in patients with diagnosed uterine cancer (1.01 ± 0.05 µmol/L) [26]. H. Sundström et al. also determined the level of selenium in the blood serum in 44 patients diagnosed with cancer of the genital organs (uterus, ovary, vulva) and in 56 patients from the control group. The mean concentration of selenium in women diagnosed with cancer of the genital organs was 1.15 ± 0.04 µmol/L; *p*-value < 0.05, and in the control group was 1.25 ± 0.03 µmol/L [27]. In another study, authors reported the level of selenium in the blood serum in 35 patients with endometrial cancer, 30 patients with ovarian cancer, and 25 women with cervical cancer, compared with 32 women in the control group. In patients with diagnosed endometrial cancer, the selenium level was lower than in the control group (1.14 ± 0.04 /µmol/L vs, 1.26 ± 0.03 µmol/L, respectively; *p*-value < 0.05) [28]. The three studies above show that in women with endometrial cancer, the level of selenium was lower than in the control group, showing the same relationship as in our studies.

In 1990, P. Knekt et al. investigated the relationship between the level of selenium in the blood serum and the risk of cancer among women and men. In women diagnosed with cancer of the reproductive organs, the selenium level was higher than in healthy patients from the control group [29]. In 2007, K. Piekutowski et al. investigated the antioxidant role of selenium in the pathogenesis of female genital cancer. The study group consisted of 50 women with diagnosed cancer of the cervix, endometrium, or ovary, and 49 patients with benign lesions of the genital organs. The control group consisted of 22 healthy women. A statistically significant lower serum selenium level was obtained in the study group [30]. In 2009, a prospective study of the level of selenium in the blood serum and whole blood was carried out in 126 patients with endometrial cancer after surgical treatment and before radiotherapy. In 105 patients, i.e., 83.3%, the selenium level was lower than recommended (*p*-value < 0.001) [31]. In 2012, the consumption of antioxidant products, including selenium, was examined in New Jersey in 417 patients diagnosed with endometrial cancer and 395 healthy women. Reduced risk of developing endometrial cancer was observed with increasing dietary selenium intake. However, the data did not reach statistical significance [32]. The 2019 analysis by Kho et al. includes 12,906 women diagnosed with endometrial cancer and 108,979 women in the control group. There was no correlation between the level of selenium in the blood and the risk of endometrial cancer [33].

In our study, we found a strong correlation between lower selenium levels and the incidence of endometrial cancer. The role of selenium in the body and the functions of selenoproteins indicate that this element is potentially important in carcinogenesis. It is difficult to explain why, in some studies, the differences in serum selenium levels between endometrial cancer patients and healthy women were not significant, or even inverse correlations were observed. Perhaps population variations are involved, including modifier genes that may influence the relevance of the level of selenium, in a particular population, to endometrial cancer risk. The influence of environmental factors should also be taken into account. For example, a diet rich in selenium, common in a given population, could cause the differences between the group of cancer patients and the healthy group to be insignificant. In extreme conditions, such a diet could be associated with an increased risk of cancer.

Women who may be deficient in selenium may be deficient in other micronutrients, particularly as selenium in the blood is dependent on selenium in the food eaten and that selenium-deficient food may also be low in other micronutrients. Therefore, future studies need to control for other micronutrients that may contribute to disease. Supplementing with selenium may fail if the real reason for disease is that the level of another micronutrient is low.

When examining the influence of selenium levels on the development of endometrial cancer, one should be aware of numerous risk factors influencing the development of the disease. During the review of the literature, we observed numerous studies showing the influence of age, menopause, coexistence of hypertension, diabetes, early first menstruation, non-fertility, and BMI on the development of endometrial cancer [34,35,36,37,38,39,40,41]. For other risk factors, i.e., contraception, hormone-replacement therapy, endometriosis, smoking, and hypothyroidism, the publications we analyzed did not provide conclusive results [42,43,44,45]. Risk factors can significantly interfere with the final result in the context of how selenium levels affect the development of endometrial cancer. Hence, the multivariate research that was included in our work is important.

Taking into account the aforementioned doubts, it should be stated that in the Polish population, a low selenium level correlates with a higher incidence of endometrial cancer and patients with low levels should constitute a group of increased risk, requiring appropriate preventive examinations. The question that remains is whether selenium supplementation in a population such as Poland may reduce the incidence of endometrial cancer. In order to answer this question, it is necessary to conduct appropriate clinical trials.

Research on the influence of selenium levels on the development of endometrial cancer is not clear. Similar controversies exist with other cancers. The literature shows a decreased level of selenium in patients diagnosed with breast cancer. Among the 18 case-control studies analyzed, which involved a total of 3374 women diagnosed with breast cancer and 3582 women from the control group, the difference in selenium level between the subgroups was 1.14 µg/L (*p*-value < 0.001) [46]. In the case of the remaining neoplasms, the results remain inconclusive and require further research [47,48].

## 5. Conclusions

A strong correlation between the level of selenium in the blood serum and the incidence of endometrial cancer indicates that patients with low levels should be a candidate group requiring appropriate preventive examinations. Further research on a larger group of patients is required. Appropriate clinical trials are necessary to determine the optimal level of selenium in the blood serum to protect against the development of endometrial cancer.

## Figures and Tables

**Table 1:** Clinical and lifestyle characteristics of patients affected with endometrial cancer and healthy controls included in the study.

Characteristics	Overall = 306 ^1^	Controls = 153 ^1^	Cases = 153 ^1^	*p*-Value
Age at sample (mean, range)	(63.67)	(63.64)	(63.71)	
	34–88	34–88	34–85	0.3
Menopause				
no	24 (7.8%)	13 (8.5%)	11 (7.2%)	
yes	282 (92%)	140 (92%)	142 (93%)	0.5
Contraception				
no	286 (93%)	140 (92%)	146 (95%)	
yes	20 (6.5%)	13 (8.5%)	7 (4.6%)	0.12
Hormone replacement therapy				
no	283 (92%)	148 (97%)	135 (88%)	
yes	23 (7.5%)	5 (3.3%)	18 (12%)	0.011
Endometriosis				
no	272 (89%)	141 (92%)	131 (86%)	
yes	34 (11%)	12 (7.8%)	22 (14%)	0.074
Hypertension				
no	132 (43%)	73 (48%)	59 (39%)	
yes	174 (57%)	80 (52%)	94 (61%)	0.073
Diabetes				
no	249 (81%)	133 (87%)	116 (76%)	
yes	57 (19%)	20 (13%)	37 (24%)	0.014
Smoker				
no	272 (89%)	140 (92%)	132 (86%)	
yes	34 (11%)	13 (8.5%)	21 (14%)	0.2
First menstruation (age)	10.00–61.00 (14.31)	11.00–20.00 (14.16)	10.00–61.00 (14.46)	0.5
Number of births	0.00–8.00	0.00–8.00	0.00–7.00	0.033
	(2.61)	(2.77)	(2.44)	
Breastfeeding				
no	78 (25%)	26 (17%)	52 (34%)	
yes	228 (75%)	127 (83%)	101 (66%)	0.002
Hypothyroidism				
no	261 (85%)	130 (85%)	131 (86%)	
yes	45 (15%)	23 (15%)	22 (14%)	>0.9
BMI	18.03–56.50 (29.38)	18.03–43.51 (27.50)	19.63–56.50 (31.27)	<0.001

^1^ n (%); Range (Mean).

**Table 2:** Results of univariate and multivariate conditional logistic regression.

	Univariable Conditional Logistic Regression			Multivariable Conditional Logistic Regression		
Characteristic	OR ^1^	95% CI ^1^	*p*-Value	OR ^1^	95% CI ^1^	*p*-Value
Selenium level (µg/L)/µmol/L						
I 8.85–57.28 (46.95; 0.59)	22.0	7.94, 61.2	<0.001	17.8	5.20, 59.0	<0.001
II 57.35–69.76 (63.60; 0.80)	5.94	2.59, 13.6	<0.001	6.40	2.15, 19.0	<0.001
III 69.90–80.45 (75.05; 0.95)	2.03	0.91, 4.53	0.084	1.42	0.49, 4.16	0.5
IV 80.64–170.76 (93.12; 1.18)	—	—		—	—	
Age	1.22	0.82, 1.82	0.3	1.27	0.61, 2.65	0.5
Menopause						
no	—	—		—	—	
yes	1.67	0.40, 6.97	0.5	0.50	0.04, 5.68	0.6
Contraception						
no	—	—		—	—	
yes	0.40	0.13, 1.28	0.12	0.57	0.09, 3.67	0.6
Hormone replacement therapy						
no	—	—		—	—	
yes	3.60	1.34, 9.70	0.011	2.76	0.59, 12.8	0.2
Endometriosis						
no	—	—		—	—	
yes	2.00	0.94, 4.27	0.074	1.50	0.44, 5.15	0.5
Hypertension						
no	—	—		—	—	
yes	1.61	0.96, 2.71	0.073	1.39	0.57, 3.36	0.5
Diabetes						
no	—	—		—	—	
yes	2.21	1.18, 4.16	0.014	1.66	0.58, 4.75	0.3
Smoker						
no	—	—		—	—	
yes	1.67	0.81, 3.41	0.2	0.87	0.24, 3.18	0.8
First menstruation (age)	1.03	0.95, 1.13	0.5	1.14	0.89, 1.47	0.3
Number of births	0.82	0.69, 0.99	0.033	0.92	0.66, 1.27	0.6
Breastfeeding						
no	—	—		—	—	
yes	0.42	0.25, 0.72	0.002	0.35	0.13, 0.90	0.030
Hypothyroidism						
no	—	—		—	—	
yes	0.94	0.49, 1.83	0.9	1.01	0.31, 3.26	>0.9
BMI	1.15	1.08, 1.21	<0.001	1.18	1.07, 1.29	<0.001

^1^ OR = Odds Ratio, CI = Confidence Interval.

## Data Availability

The data presented in this study are available on request from the corresponding authors.
